# The Effect of Dietary Protein Restriction in Phase Feeding Systems on Nitrogen Metabolism and Excretion in Pig Production

**DOI:** 10.3390/ani15111521

**Published:** 2025-05-23

**Authors:** Wiesław Sobotka, Aleksandra Drażbo

**Affiliations:** 1 Department of Animal Nutrition, Feed Science, and Cattle Breeding, University of Warmia and Mazury in Olsztyn, Oczapowskiego 5, 10-850 Olsztyn, Poland; 2 Department of Poultry Science and Apiculture, University of Warmia and Mazury in Olsztyn, Oczapowskiego 5, 10-850 Olsztyn, Poland; aleksandra.drazbo@uwm.edu.pl

**Keywords:** pigs, nutritional strategies, nitrogen metabolism, feces and urine characteristics, nitrogen excretion, environment

## Abstract

In recent years, much attention has been paid to environmental protection, not only by reducing emissions of harmful gases from industry, but also by reducing the excretion of biogenic compounds or ammonia emissions from agriculture, including animal production. The aim of this study was to determine the effects of complete diets with reduced inclusion levels of crude protein and limiting essential amino acids, fed to pigs in two- and three-phase feeding systems, and the feeding system on crude protein digestibility, nitrogen retention and utilization, fecal and urinary pH, fecal and urinary nitrogen and ammonia levels, and nitrogen excretion. Digestibility-balance trials were performed on 24 growing–finishing pigs housed in individual metabolism crates, in three groups, in two- and three-phase feeding systems. The pigs were fed the following diets: C-control diet; L-low-protein diet where the levels of crude protein and essential amino acids (lysine, methionine + cystine, threonine, and tryptophan) were reduced by 15% relative to diet C; L+AA-low-protein diet supplemented with crystalline lysine, methionine, threonine, and tryptophan to the standard levels (as in diet C). Diets L fed to pigs in two- and three-phase feeding systems significantly decreased crude protein digestibility and nitrogen retention, particularly in the three-phase system. The supplementation of diets L+AA with crystalline essential amino acids improved crude protein digestibility and nitrogen retention and utilization, especially in the two-phase system. Reduced concentrations of crude protein and essential amino acids in diets L contributed to a significant increase in feces and urine acidity in both two- and three-phase feeding systems. The supplementation of diets L+AA with essential amino acids resulted in a significant increase in urinary pH and a non-significant increase in fecal pH. This experimental factor had no effect on fecal ammonia concentration in group L+AA. The values of pH and total fecal nitrogen were somewhat higher in the two-phase system than in the three-phase system. Fecal ammonia concentration was similar in both systems. The three-phase feeding system contributed to a decrease in urinary pH and total urinary nitrogen. The analyzed feeding systems had no significant effect on urinary ammonia concentration. It was estimated that a reduction in crude protein (by 20–25 g/kg) and essential amino acid levels in pig diets, relative to the standard levels, reduced nitrogen excretion by 18.7% and 15.6% in two- and three-phase feeding systems, respectively. The supplementation of low-protein diets (L) with lysine, methionine, threonine, and tryptophan induced a further reduction in nitrogen excretion. A comparison of the effects of feeding systems (two-phase system vs. three-phase system) on crude protein digestibility and nitrogen retention and utilization revealed that better results were obtained in the two-phase feeding system.

## Simple Summary

The crude protein content of diets can be reduced, and its amount can be adjusted to meet the nutrient requirements of pigs at different stages of production to improve nitrogen utilization and reduce nitrogen excretion into the environment. In order to achieve this goal, the dietary levels of essential amino acids should be precisely balanced by selecting the most suitable feed components or by supplementing crystalline amino acids. Digestibility-balance trials were performed on growing–finishing pigs in two- and three-phase feeding systems. The pigs were fed the following diets: C-control diet; L-low-protein diet where the levels of crude protein and essential amino acids (lysine, methionine + cystine, threonine, and tryptophan) were reduced by 15% relative to diet C; L+AA-low-protein diet supplemented with crystalline lysine, methionine, threonine, and tryptophan to the standard levels (as in diet C). The effects of different inclusion levels of crude protein and limiting essential amino acids in pig diets and the feeding system on crude protein digestibility, daily nitrogen balance, fecal and urinary pH, fecal and urinary nitrogen and ammonia concentrations, and nitrogen excretion were determined in the study. It was found that the crude protein content of complete pig diets can be reduced by 20–25 g/kg (approx. 15%) in both two- and three-phase feeding systems while maintaining high protein digestibility, and high nitrogen retention and utilization. The supplementation of low-protein diets with crystalline lysine, methionine, threonine, and tryptophan improved crude protein digestibility and nitrogen balance parameters. Reduced concentrations of protein and essential amino acids in pig diets contributed to fecal and urine acidification. The supplementation of low-protein diets with essential amino acids increased fecal and urinary pH and urinary ammonia concentration. A reduction in crude protein (by 20–25 g/kg) and limiting essential amino acid levels in pig diets, relative to the standard levels, reduced nitrogen excretion by 18.7% and 15.6% in two- and three-phase feeding systems, respectively.

## 1. Introduction

In recent years, much attention has been paid to environmental protection in order to reduce harmful gas emissions from industrial sources as well as the excretion of biogenic compounds and the emissions of ammonia, methane, and odors from agricultural sources. Environmental protection is one of the key objectives of the Common Agricultural Policy (CAP), and it is particularly important as both the scale and concentration of animal production continue to increase. Environmental public goods are to be provided under the CAP as part of the non-productive tasks of common agriculture [1,2]. The environmental impact of biogenic elements such as nitrogen and phosphorus and the emissions of ammonia, greenhouse gases (GHGs), and odors have become a serious challenge in animal production in terms of livestock farming technologies and their cost intensity [3,4,5]. Nitrogen from natural fertilizers is more widely distributed in the environment than nitrogen from mineral fertilizers due to the volatilization of ammonia and nitric oxides, as well as nitrate leaching into groundwater and its incorporation into soil organic matter [6,7,8]. Nitrogen present in soil organic matter is converted to NH_4_^+^ during the mineralization process, which includes ammonization and ammonification. Amino acids are rapidly mineralized in the soil and are degraded to NH_3_ and CO_2_ within a few days. Ammonia released by mineralization is converted to N-NH_3,_ which is available to plants and is retained in the soil solution. Ammonia volatilization can occur under favorable conditions, contributing to soil acidification and eutrophication of groundwater and surface water [6,9,10]. It is estimated that the agricultural sector is responsible for global emissions of 25% CO_2_, 50% CH_4_, and 70% N_2_O, accounting for nearly 13% of total global anthropogenic GHG emissions [3].

The European Union has identified the monitoring of and reduction in nitrogen emissions from livestock farms as a strategic objective [11,12,13]. Worldwide, pig production is estimated to be responsible for approximately 15% of NH_3_ emissions associated with livestock [14]. In Europe, around 80% of NH_3_ is produced in livestock facilities [15], and pig production represents nearly 25% of livestock ammonia emissions [16]. 

Currently, various strategies are being undertaken to reduce nitrogen emissions to the environment from livestock production, including a manure management strategy through covered storage, acidification and composting with added carbon [17], a grazing and pasture management strategy through rotational grazing and animal density adjustment and integration of nitrogen-fixing legumes [18], and a nutritional strategy related to phase and precision feeding, including supplementation with tannins [19] and saponins [20].

The nitrogen content of pig excreta is not constant and can vary depending mainly on the quantity and quality of dietary protein. The quantitative and qualitative composition of nitrogen fractions in pig manure is determined by the amount and quality of dietary protein [21]. Feeding with excess protein results in the excretion of excess nitrogen. A similar effect is exerted by diets deficient in limiting essential amino acids. In the latter case, additional deamination of amino acids increases the amount of released nitrogen, and readily soluble nitrogen compounds increase the proportion of nitrogen fractions in manure [22,23]. 

Precision pig feeding contributes to reducing nitrogen excretion, and GHG and ammonia emissions [5,24]. The protein content of diets can be reduced, and the amount of dietary protein can be adjusted to meet the nutrient requirements of pigs at different stages of production [25,26,27,28]. In practice, adequate balancing of pig diets and reducing dietary protein concentration requires changes in their composition and improving nutrient digestibility.

The aim of this study was to determine the effects of complete diets with different inclusion levels of crude protein and limiting essential amino acids, fed to pigs in two- and three-phase feeding systems, and the feeding system on crude protein digestibility, nitrogen retention and utilization, fecal and urinary pH, fecal and urinary nitrogen and ammonia levels, and nitrogen excretion.

## 2. Materials and Methods 

The animal protocol and the number of animals used in the study were consistent with regulations of the Local Institutional Animal Care and Use Committee (decision No. 5/2008), Olsztyn, Poland. The study was carried out in accordance with EU Directive 2010/63/EU on the protection of animals used for scientific purposes [29]. 

### 2.1. Animals, Diets, and Experimental Protocols in Digestibility-Balance Trials

Crude protein digestibility and daily nitrogen balance were determined during digestibility-balance trials in pigs fed complete diets with different inclusion levels of crude protein and essential amino acids. The analyses were performed in the animal research laboratory of the Department of Animal Nutrition, Feed Science, and Cattle Breeding at the University of Warmia and Mazury in Olsztyn. During the trials, a six-day experimental period was preceded by an eight-day pre-experimental (adaptation) period. The experimental materials comprised 24 male crossbred [(Polish Landrace × Polish Large White ♀) × Duroc ♂] growing-finishing pigs, divided into three groups, with eight animals per group during grower *(Experiment 1)* and finisher *(Experiment 2)* phases in a two-phase feeding system, and during grower 1 *(Experiment 1A*), grower 2 *(Experiment 1B*), and finisher *(Experiment 2A)* phases in a three-phase feeding system, according to the experimental design presented in Table 1.

In the two-phase feeding system, digestibility-balance trials were conducted using the balance method for grower and finisher diets fed to growing-finishing pigs with a body weight of 30–70 kg and 70–110 kg, respectively (*Experiments 1 and 2*, Table 2).

In the three-phase feeding system, digestibility-balance trials were conducted for grower 1, grower 2, and finisher diets fed to growing–finishing pigs with a body weight of 30–55 kg, 55–80 kg, and 80–110 kg, respectively *(Experiments 1A, 1B, and 2A*, Table 2). The animals were housed in individual metabolism crates with a surface area of 1.2 m^2^ (150 cm in length, 70 cm in width) and a height of 80 cm, with a slatted floor, equipped with feed troughs and automatic drinkers. Feed was rationed, and the concentrations of crude protein and limiting essential amino acids in diets are presented in Table 1. The daily ration was determined based on the Nutrient Requirements of Pigs [30], assuming an average daily gain of 850 g. Pigs were fed crumbled feed that was offered wet (feed/water ratio of 1:1) twice daily at 7.00 a.m. and 2.00 p.m., and had free access to water.

During the six-day experimental period of each digestibility-balance trial *(Experiments 1, 2, 1A, 1B, and 2A*), feces and urine were collected quantitatively. A 5% sample, taken from the feces produced during 24 h, was frozen. At the end of each experiment, nitrogen content was determined in the average dried fecal sample. Urine was collected in plastic containers with the addition of sulfuric acid to maintain pH below 2.0, and 5% samples were collected to determine nitrogen content [31]. The apparent digestibility coefficient of crude protein was calculated based on the protein content of feces and diets, and protein intake and output, using the following equation: AD (%) = I − O/I × 100, where AD—apparent digestibility coefficient (%); I—nutrient intake (g); O—fecal nutrient output (g); O/I—nutrient digested (g).

Nitrogen retention was determined based on dietary nitrogen intake and fecal and urinary nitrogen excretion, depending on the feeding system and the concentrations of limiting essential amino acids in pig diets. Nitrogen utilization was calculated based on the apparent biological value of protein (PBV) expressed as nitrogen retained relative to nitrogen digested.

### 2.2. Selected Serum Biochemical Parameters

Blood samples were collected for analysis from eight animals per group to determine indicators of selected biochemical processes in pigs fed diets with different inclusion levels of crude protein and essential amino acids in two- and three-phase feeding systems. The samples were collected from the anterior vena cava of live animals at the end of the experimental period in each digestibility-balance trial *(Experiments 1 and 2, and Experiments 1A, 1B, and 2A)*. Whole blood samples were collected into test tubes containing a chemically neutral clotting activator. The samples were allowed to clot at room temperature for 30 min, and they were then centrifuged in the MPW-348 centrifuge at 2000 rpm for 10 min. Serum samples were stored at −20 °C until analysis. Protein metabolism was analyzed based on serum total protein and ammonium nitrogen levels. Liver function was evaluated based on the activities of aspartate aminotransferase (AST) and alanine aminotransferase (ALT). 

### 2.3. Environmental Impact of Diets Fed to Pigs in Two- and Three-Phase Feeding Systems

The environmental impact of diets fed to pigs in two- and three-phase feeding systems was determined by analyzing fecal and urine samples and the amount of nitrogen excreted into the environment, taking into account the experimental factors (Table 1). Feces and urine were characterized based on acidity (pH), total nitrogen content, and ammonia content. Fecal and urinary pH values were determined in fresh samples during the experimental period of digestibility-balance trials. The content of total nitrogen and ammonia was determined in frozen fecal samples and urinary samples preserved with sulfuric acid. 

Fecal and urinary nitrogen excretion, depending on the concentrations of crude protein and limiting essential amino acids in pig diets and feeding system, was estimated mathematically based on nitrogen balance in pigs fed grower and finisher diets in the two-phase feeding system and grower 1, grower 2, and finisher diets in the three-phase feeding system.

### 2.4. Chemical Analyses

Crude protein concentration in diets and fecal and urinary nitrogen content were determined by standard methods [31]. The amino acid composition of protein in feedstuffs was determined using an AAA 400 automatic amino acid analyzer. The samples were hydrolyzed with 6M HCL at 110 °C for 24 h. The concentrations of sulfur-containing amino acids were determined after sample oxidation with performic acid. Tryptophan content was determined in accordance with Polish Standard PN-77/R-64820:1997 [32]. The results were used to calculate the concentrations of lysine, methionine + cystine, threonine, and tryptophan in diets.

Fecal and urinary pH (acidity) was determined in fresh samples using a Hanna Instruments pH-meter (model 301) and a microelectrode. Total nitrogen content was determined by the Kjeldahl method [31], and ammonia content was determined by the Conway method according to Industry Standard BN-74/9162-01 [33].

Serum ammonium nitrogen levels were determined by the kinetic method. Total serum protein was determined by the biuret method. The activities of AST and ALT were evaluated by the kinetic method using the CORMAY ACCENT-200 automatic biochemistry analyzer and commercial Cormay kits. The results were expressed in SI units [34].

### 2.5. Statistical Analysis

The results of digestibility-balance trials were processed statistically by one-way analysis of variance (ANOVA) using licensed STATISTICA 13.0 software. The significance of differences between group means was determined by Duncan’s multiple range test at significance levels of *p* ≤ 0.05 and *p* ≤ 0.01 [35].

## 3. Results

### 3.1. Crude Protein Digestibility, and Nitrogen Retention and Utilization

An analysis of the two-phase feeding system (Table 3) revealed that a reduction in the inclusion levels of crude protein and total lysine (the first limiting amino acid in pig diets), methionine+cystine, threonine, and tryptophan in diets L highly significantly decreased protein digestibility, from 82.1% in the control group C to 79.4% in group L. The supplementation of low-protein diets (L+AA) with crystalline lysine, methionine, threonine, and tryptophan highly significantly improved crude protein digestibility, which reached 82.4% in group L+AA. 

Changes in crude protein digestibility followed a similar pattern in pigs fed diets with different concentrations of crude protein and limiting essential amino acids in the three-phase feeding system (Table 3), where the apparent digestibility coefficient of crude protein was determined at 82.2%, 80.6%, and 81.6%, respectively. The differences between group means (C vs. L and L vs. L+AA) were significant. The other experimental factor, feeding system (the two-phase system vs. the three-phase system), had no significant effect on the apparent digestibility coefficient of crude protein, which ranged from 81.3% to 81.5% (Table 3). 

As shown in Table 3, fecal nitrogen excretion was not significantly affected by reduced concentrations of crude protein and limiting essential amino acids in diets L in any of the feeding systems. The average values of this parameter were 12.6/12.1 g/kg^−1^ (group C) vs. 12.5/11.4 g/kg^−1^ (group L). The supplementation of diets L+AA with crystalline lysine, methionine, threonine, and tryptophan induced a further significant reduction in fecal nitrogen excretion. The average values of this parameter were 10.8/10.7 g/kg^−1^ (group L+AA) vs. 12.5/11.4 g/kg^−1^ (group L). The feeding system had no significant effect on fecal nitrogen excretion. 

In both two- and three-phase feeding systems, diets L with decreased crude protein content highly significantly reduced urinary nitrogen excretion, relative to control diets C (Table 3). The values of this parameter were 29.8/28.3 g/kg^−1^ vs. 21.2/22.7 g/kg^−1^. The supplementation of diets L+AA with crystalline lysine, methionine, threonine, and tryptophan had no significant effect on urinary nitrogen excretion relative to diets L. Urinary nitrogen excretion was not affected by this experimental factor in the two analyzed feeding systems, either. The average values of this parameter ranged from 24.3 g/kg^−1^ to 24.6 g/kg^−1^.

The experimental factor, including the inclusion levels of both crude protein and amino acids (lysine, methionine + cystine, threonine, and tryptophan) in pig diets, had no significant effect on nitrogen retention (Table 3). Daily nitrogen retention was lowest in pigs receiving low-protein diets L in both two- and three-phase feeding systems, compared with pigs fed control diets C, and the values of this parameter were 27.2/24.8 g/kg^−1^ vs. 28.2/27.5 g/kg^−1^. The supplementation of low-protein diets L+AA with crystalline lysine, methionine, threonine, and tryptophan did not significantly increase daily nitrogen retention in pigs, which was determined at 27.7 g/kg^−1^ and 24.7 g/kg^−1^; a more satisfactory result was achieved in the two-phase feeding system.

An analysis of the apparent biological value of protein expressed as nitrogen retained relative to nitrogen digested revealed that it improved significantly in response to a 15% reduction in the crude protein content of pig diets, especially in the two-phase feeding system. The lowest utilization of digested nitrogen was observed in pigs fed diets C with standard concentrations of crude protein and limiting essential amino acids (48.6% in the two-phase feeding system and 49.3% in the three-phase feeding system). Pigs receiving diets L, in which crude protein content was reduced by 15% relative to control diets, were characterized by significantly higher utilization of digested nitrogen (increase from 48.6% to 56.2% in the two-phase feeding system, and from 49.3% to 52.2% in the three-phase feeding system). The supplementation of low-protein diets L+AA with limiting essential amino acids had no significant effect on a further improvement in the utilization of digested nitrogen in the analyzed feeding systems. 

Serum biochemical parameters, reflecting nitrogen metabolism, are presented in Table 4. The experimental factor, i.e., different dietary inclusion levels of crude protein and limiting essential amino acids, had a significant effect on serum urea levels in pigs, especially in the two-phase feeding system, pointing to differences in nitrogen utilization. Regardless of the feeding system, the level of this metabolite was highest in pigs fed diets C with standard quantity and quality of crude protein (29.02 g/dL and 25.44 g/dL). A 15% reduction in the crude protein content of diets L significantly decreased serum urea levels (to 27.01 g/dL and 23.00 g/dL). The supplementation of low-protein diets L+AA with crystalline lysine, methionine, threonine, and tryptophan induced a further significant decrease in the level of this metabolite, especially in the two-phase feeding system (24.73 g/dL). This relationship was not observed in pigs fed diets L+AA in the three-phase feeding system, where serum urea concentration was 24.64 g/dL.

An analysis of total serum protein in pigs fed diets with different inclusion levels of crude protein and limiting essential amino acids in two- and three-phase feeding systems (Table 4) revealed significant differences between groups, especially in the three-phase feeding system, and the noted values ranged from 63.9 g/L to 67.5 g/L. The activities of AST and ALT in the blood serum of pigs varied significantly depending on the dietary inclusion levels and quality of crude protein. Serum ALT concentration was highest in pigs fed diets L with reduced crude protein content in both two- and three-phase feeding systems (47.75 U/L and 49.00 U/L, respectively). The addition of crystalline lysine, methionine, threonine, and tryptophan had a greater effect on serum ALT concentration, which decreased to 43.31 U/L in pigs receiving diet L+AA in the two-phase feeding system. Pigs fed diets C with standard quantity and quality of crude protein were characterized by significantly higher serum ALT concentration (46.10 U/L–46.20 U/L) than those fed low-protein diets L. An analysis of the effect of the experimental factors on serum AST concentration revealed that reduced inclusion levels of crude protein and limiting essential amino acids in diets L contributed to a significant increase in the concentration of this metabolite from 32.96 U/L to 35.21 U/L, especially in the three-phase feeding system. The supplementation of diets L with limiting essential amino acids induced a further significant increase in serum ALT concentration in both two- and three-phase feeding systems (from 23.88 U/L to 35.38 U/L and from 35.21 U/L to 37.71 U/L, respectively). 

### 3.2. Feces and Urine Characteristics

The inclusion levels of crude protein and limiting essential amino acids in diets fed to pigs in two- and three-phase feeding systems affected the analyzed physical properties of feces (Table 5). Reduced concentrations of crude protein and limiting essential amino acids in diets L contributed to significantly higher acidity (lower pH) in feces, especially in the two-phase feeding system, compared with control diets C. 

The supplementation of low-protein diets L+AA with limiting essential amino acids (crystalline lysine, methionine, threonine, and tryptophan) to the standard levels as in diets C increased fecal pH from 6.16/6.17 to 6.29/6.23, but the increase was not significant. Pigs fed diets L+AA with reduced crude protein concentration and enriched with limiting essential amino acids were characterized by the lowest total fecal nitrogen concentration (0.73% and 0.71%), most likely due to a more favorable protein balance and higher crude protein digestibility in diets L+AA. This experimental factor had no effect on fecal ammonia concentration in group L+AA. 

The physical parameters of fecal samples collected from pigs in two- and three-phase feeding systems did not differ significantly. Fecal pH and total fecal nitrogen concentration were somewhat higher in the two-phase system than in the three-phase system (6.28 vs. 6.24 and 0.77% vs. 0.73%, respectively). Fecal ammonia concentration was similar in both systems (0.04% vs. 0.05%).

Changes in urine parameters, particularly pH, followed a similar pattern to feces parameters (Table 6). Urine acidity increased highly significantly in pigs fed low-protein diets L in the three-phase feeding system. The supplementation of diets L+AA with limiting essential amino acids caused a significant increase in urinary pH (from 6.31/6.41 to 6.46/6.41). 

Differences in urinary ammonia concentration were found, especially in the two-phase feeding system, but this parameter was not significantly lower in pigs fed diet L than in those fed diet C (0.37% vs. 0.40%); such a relationship was not observed in the three-phase feeding system. The supplementation of diets L+AA with lysine, methionine, threonine, and tryptophan increased urinary ammonia concentration, particularly in the two-phase feeding system, but the noted increase was not significant, and this parameter reached the level noted in the control group C. The three-phase feeding system contributed to a decrease in urinary pH and total urinary nitrogen concentration, whereas urinary ammonia concentration was not affected by the feeding system.

### 3.3. Fecal and Urinary Nitrogen Excretion

The effects of the concentrations of crude protein and limiting essential amino acids in pig diets and the feeding system on nitrogen excretion are presented in Table 7. It was estimated that regardless of the feeding system, nitrogen intake was highest in pigs fed control diets C with the highest crude protein content (987.7 g/kg^−1^ in the two-phase system and 950.1 g/kg^−1^ in the three-phase system), and lowest in those fed diets L+AA with reduced crude protein content and with the addition of crystalline lysine, methionine, threonine, and tryptophan (847 g/kg^−1^ in the two-phase system and 816.7 g/kg^−1^ in the three-phase system.

Fecal and urinary nitrogen excretion was highest in pigs fed diets C with the standard quantity and quality of crude protein. In group C, fecal nitrogen excretion was 176.4 g/kg^−1^, urinary nitrogen excretion was 416.57 g/kg^−1^, and total nitrogen excretion was 592.9 g/kg^−1^ in the two-phase feeding system, and the corresponding values in the three-phase system were 168.97 g/kg^−1^, 396.77 g/kg^−1^, and 565.67 g/kg^−1^, respectively.

Based on the above nitrogen balance data, nitrogen excretion into the environment was estimated in pigs fed diets with different concentrations of crude protein and limiting essential amino acids. In the two-phase feeding system (Table 7), total nitrogen excretion was highest in the control group C (529.87 g/kg^−1^). A 15% reduction in the levels of crude protein and total lysine in diets L reduced total nitrogen excretion by 110.6 g/kg^−1^ (18.7%). The supplementation of diets L+AA with crystalline lysine, methionine, threonine, and tryptophan induced a further reduction in total nitrogen excretion, by 23.1 g/kg^−1^ (4.8%) relative to group L. An analysis of the three-phase feeding system (Table 7) revealed that, similarly to the two-phase system, nitrogen excretion was highest in control group pigs (565.67 g/kg^−1^). The adjustment of the crude protein content of diets to the physiological status of pigs improved nitrogen utilization in animals fed diets L+AA supplemented with crystalline lysine, methionine, threonine, and tryptophan, resulting in the lowest nitrogen excretion (477.4 g/kg^−1^). Diets L+AA with decreased crude protein content reduced nitrogen excretion by 88.2 g/kg^−1^ (15.6%), whereas their supplementation with crystalline essential amino acids did not induce a further significant reduction in fecal or urinary nitrogen excretion. Nitrogen excretion was not significantly affected by the feeding system (Table 7), and it was only 7 g/kg^−1^ (1.4%) lower in the three-phase system than in the two-phase system. 

## 4. Discussion

According to some authors [36,37], protein digestibility increases with its increasing dietary inclusion levels, which is related to the higher digestibility of supplemental protein ingredients. In the present study, the soybean meal content of pig diets was reduced to decrease crude protein concentration. As a result, crude protein digestibility decreased from 2.7% to 1.6% in both two- and three-phase feeding systems. Another study [38] demonstrated that crude protein digestibility was also affected by the levels of crude protein and amino acids in pig diets, and the apparent digestibility coefficient of crude protein exceeded 80%. In turn, when the protein content of grower pig diets was reduced from 16% to 12%, the apparent digestibility coefficient of crude protein decreased by 5.3% [39]. Other authors reported that diets with reduced crude protein content, supplemented with essential amino acids, had no negative effect on nutrient digestibility. Nutrient digestibility coefficients were comparable in diets containing 14% protein, enriched with lysine, methionine, threonine, and tryptophan, and in diets containing 16.5% protein [40]. In the current study, low-protein diets containing 14.3% and 13.5% crude protein in two- and three-phase feeding systems, respectively, were supplemented with limiting essential amino acids, which increased crude protein digestibility to the levels noted in diets containing 16.6% and 15.9% crude protein, respectively. It appears that amino acid supplementation improved not only the digestibility of crude protein, but also its apparent biological value, as manifested by reduced fecal and urinary nitrogen excretion. 

Shriver et al. [41] found that the supplementation of a low-protein diet with lysine, methionine, threonine, tryptophan, isoleucine, and valine reduced fecal and urinary nitrogen excretion in finishing pigs. Figueroa et al. [42] added synthetic amino acids (lysine, methionine, threonine, and tryptophan) to a diet whose protein content was decreased from 17.7% to 14%, which reduced nitrogen excretion by approximately 30%. In the work of Cahn et al. [43], dietary crude protein reduction from 16.5% to 12.5%, with amino acid supplementation as in the control group, did not significantly affect fecal nitrogen excretion, whereas urinary nitrogen excretion was reduced by around 45% in growing-finishing pigs.

In the experiments conducted by Kerr et al. [44] and Leek [45], nitrogen excretion in growing–finishing pigs was reduced by 8.4% and 8.7%, respectively, for every 10 g/kg decrease in dietary crude protein content. In the present study, a reduction (by 20–25 g/kg) in the dietary inclusion levels of crude protein in pig diets in two- and three-phase feeding systems significantly reduced nitrogen excretion, particularly urinary nitrogen excretion, by 18.7% in the two-phase system and by 15.6% in the three-phase system. The supplementation of low-protein diets with limiting essential amino acids (lysine, methionine, threonine, and tryptophan) did not induce a further significant reduction in urinary nitrogen excretion. Total nitrogen excretion decreased by 4.8% in the two-phase system and by 1.7% in the three-phase system. The reduction in urinary nitrogen excretion is correlated with a decrease in the quantity and quality of crude protein in pig diets. It should be assumed that in the two-phase feeding system, pig diets with reduced protein content were characterized by a slightly better balance of protein in essential amino acids than the diets used in the three-phase feeding system. Animals fed high-protein diets are unable to store excess protein in muscle tissue; therefore, it is excreted, primarily in the urine. The results of this study corroborate the findings of other authors [41,46] who found that urinary nitrogen excretion decreased by an average of 11–25% in pigs fed low-protein diets supplemented with essential amino acids. Similar observations were made by Patras et al. [47] and Cappelaere et al. [48].

In the current study, a 15% reduction in the crude protein content of pig diets improved the apparent biological value of protein in both two- and three-phase feeding systems. Dietary supplementation with lysine, methionine, threonine, and tryptophan also exerted a positive effect on this parameter. The apparent biological value of protein increased by 2% when the crude protein content of pig diets was reduced from 18% to 14%, and crystalline lysine, methionine, threonine, and tryptophan were added to the low-protein diet. In the present study, a reduction in crude protein concentration in pig diets in the three-phase feeding system, combined with essential amino acid supplementation, improved nitrogen balance. Similar results were reported by other authors [49,50,51] who found that nitrogen retention decreased by 12% in young pigs fed a diet containing 15.6% protein, compared with those receiving a diet containing 18.5% protein. Nitrogen retention decreased by only 7% when the animals had unlimited access to feed. In another study [50], a reduction in crude protein concentration from 18% to 15%, with simultaneous dietary supplementation with lysine, methionine, threonine, and tryptophan, reduced daily nitrogen retention in growing pigs by 7%. Lower nitrogen retention in growing–finishing pigs fed low-protein diets supplemented with limiting essential amino acids may be due to several reasons, including insufficient intake of one or more essential amino acids, differences in the digestibility of these amino acids, or their inadequate balance. An important role is also played by endogenous nitrogen, which is recycled in large quantities in pigs, while its digestibility is lower than that of other nutrients [52]. Previous research [46,53] has shown that endogenous nitrogen losses, which are closely related to total nitrogen losses, increase with decreasing dietary crude protein concentrations. Therefore, higher endogenous nitrogen losses may reduce the utilization of nitrogen from low-protein diets. 

## 5. Conclusions

The results of this study indicate that the crude protein content of complete diets for growing–finishing pigs can be reduced by 20–25 g/kg (approx. 15%), relative to the standard level, in both two- and three-phase feeding systems, while maintaining high protein digestibility, and high nitrogen retention and utilization. The supplementation of low-protein diets with crystalline lysine, methionine, threonine, and tryptophan improves crude protein digestibility and nitrogen retention and utilization. A 15% reduction in the dietary inclusion levels of crude protein and essential amino acids, relative to the standard levels in pig diets, reduces nitrogen excretion by 18.7% and 15.6% in two- and three-phase feeding systems, respectively. The supplementation of low-protein diets with lysine, methionine, threonine, and tryptophan induced a further reduction in nitrogen excretion by 4.8%, especially in the two-phase feeding system. The effects of feeding systems (two-phase system vs. three-phase system) on crude protein digestibility and nitrogen retention and utilization revealed that better results were obtained in the two-phase feeding system.

## Figures and Tables

**Table 1:** Experimental design.

Experimental Factor	Pig Feeding System					
	Two-Phase System			Three-Phase System		
	Diet ^1^					
	C	L	L+AA	C	L	L+AA
Reduced levels of crude protein and amino acids (%)	0	15	15+AA	0	15	15+AA
Dietary levels of crude protein (%) and essential amino acid (%) during the entire fattening period ^2^						
Crude protein	16.60	14.20	14.30	15.90	13.50	13.50
Lysine	0.91	0.78	0.91	0.84	0.72	0.84
Methionine + cystine	0.69	0.59	0.69	0.64	0.55	0.64
Threonine	0.59	0.50	0.59	0.56	0.46	0.56
Tryptophan	0.21	0.18	0.21	0.19	0.16	0.19
Number of pigs (head)	8	8	8	8	8	8

^1^ C—control diet with standard crude protein content; L-low-protein diet where the levels of crude protein and essential amino acids (lysine, methionine + cystine, threonine, and tryptophan) were reduced by 15% relative to diet C; L+AA-low-protein diet supplemented with crystalline lysine, methionine, threonine, and tryptophan to the standard levels (as in diet C). ^2^ concentrations of crude protein, lysine, methionine + cystine, threonine, and tryptophan are arithmetic means calculated for *Experiments 1 and 2* in the two-phase feeding system and *Experiments 1A, 1B, and 2A* in the three-phase feeding system.

**Table 2:** Composition and nutritional value of diets fed to pigs in two- and three-phase feeding systems.

Item	Two-Phase Feeding System			Three-Phase Feeding System		
	Diet ^1^					
	Grower/Finisher Fed to Pigs with a Body Weight of 30–70 kg (*Experiment 1*)/70–110 kg (*Experiment 2)*			Grower 1/Grower 2/Finisher Fed to Pigs with a Body Weight of 30–55 kg (*Experiment 1A*)/55–80 kg (*Experiment 1B*)/80–110 kg (*Experiment 2A)*		
	C	L	L+AA	C	L	L+AA
Reduced levels of crude protein and amino acids	0	15	15 +AA	0	15	15 +AA
Feed components (%)						
Ground grain (wheat + barley)	76.8/83.6	81.9/89.2	81.7/88.8	76.8/83.3/87.8	82.4/89.2/95.4	81.5/88.8 /95.5
SBM ^2^	15/6	9/0	9/-	15/9/-	9/3/-	9/3/-
RSM “00” ^3^	5/8	5/8	5/8	5/5/10	5/5/2	5/5/2
Rapeseed oil	-	0.8/0.4	0.8/0.4	-/0.3/-	0.8/0.4	0.8/0.4
Mineral–vitamin premix ^4^	3.0/2.2	3.0/2.2	3.0/2.2	3.0/2.2/2.0	3.0/2.2/2.0	3.0/2.2/2.0
Supplementation with crystalline amino acids (%):						
L-lysine HCL (78%)	0.20/0.23	0.25/0.23	0.41/0.42	0.20/ 0.23/0.2	0.25/0.28 /0.2	0.41/0.42 /0.35
DL-methionine (99%)	-	-	0.15/0.05	-	-	0.15/0.09/ 0.06
L-threonine (98%)	-	-	0.09/0.09	-	-	0.09/0.09 /0.11
DL-tryptophan (98%)	-	-	0.03/0.04	-	-	0.03/03.03
Average nutritional value of diets during the entire fattening period (g/kg^−1^):						
Crude protein	165	140	141	158	135	136
Digestible protein	135	111	116	130	109	111
Lysine	6.9	5.9	6.9	6.6	6.0	6.6
Methionine + cystine	6.9	5.9	6.9	6.6	6.0	6.6
Threonine	5.9	5.0	5.9	5.6	4.7	5.6
Tryptophan	2.0	1.7	2.0	1.9	1.7	1.9
Crude fiber	40.1	39.6	39.4	42.1	41.3	43.0
Metabolizable energy (MJ kg^−1^) ^5^	13.11	13.04	13.11	13.16	13.12	13.10

^1^ see Table 1; ^2^ soybean meal; ^3^ rapeseed meal “00”; ^4^ limestone (1.0/0.7); dicalcium phosphate (0.7/0.5); salt (0.3/0.3); mineral–vitamin premix (1.0/0.7); limestone (0.8/0.7/0.6); dicalcium phosphate (0.7/0.7/0.3); salt (0.3/0.3/0.25) ^5^ values calculated based on own results (unpublished data, Sobotka, Drażbo 2019) and the Nutrient Requirements of Pigs (2014).

**Table 3:** Protein digestibility, nitrogen retention and utilization in pigs fed diets with different inclusion levels of crude protein and limiting essential amino acids in two- and three-phase feeding systems.

Item	Two-Phase Feeding System				Three-Phase Feeding System			
	Group ^1^							
	C	L	L+AA	*p*-Value	C	L	L+AA	*p*-Value
Reduced levels of crude protein and amino acids (%)	0	15	15 +AA		0	15	15 +AA	
different inclusion levels of crude protein and limiting essential amino acids								
N digestibility × 6.25 (%)	82.1 ^B^	79.4 ^A^	82.4 ^B^	0.000	82.2 ^b^	80.6 ^a^	81.6 ^ab^	0.042
Daily nitrogen balance (g kg^−1^):								
N intake	70.6 ^B^	60.9 ^A^	60.5 ^A^	0.000	67.9 ^B^	58.9 ^A^	58.3 ^A^	0.000
Fecal N excretion	12.6 ^B^	12.5 ^B^	10.8 ^A^	0.002	12.1 ^B^	11.4 ^b^	10.7 ^Aa^	0.011
N digested	58.0 ^B^	48.4 ^A^	49.7 ^A^	0.000	55.8 ^B^	47.5 ^A^	47.6 ^A^	0.000
Urinary N excretion	29.8 ^B^	21.2 ^A^	22.0 ^A^	0.000	28.3 ^B^	22.7 ^A^	22.9 ^A^	0.000
N retention	28.2	27.2	27.7	0.805	27.5	24.8	24.7	0.066
Nitrogen utilization (%):								
N retention/N intake	39.9 ^a^	44.7 ^b^	45.8 ^b^	0.033	40.5	42.1	42.4	0.597
N retention/N digested	48.6 ^A^	56.2 ^B^	55.7 ^B^	0.014	49.3	52.2	51.9	0.433
different feeding systems								
N digestibility × 6.25 (%)	81.3				81.5			0.857
Daily nitrogen balance (g kg^−1^):								
N intake	64.0				61.7			0.061
Fecal N excretion	12.0				11.4			0.064
N digested	52.0				50.3			0.121
Urinary N excretion	24.3				24.6			0.947
N retention	27.5 ^b^				25.7 ^a^			0.021
Nitrogen utilization (%):								
N retention/N intake	42.9				41.6			0.179
N retention/N digested (PBV) ^2^	53.7				51.1			0.176

^1^—explanation as in Table 1; ^2^—apparent biological value of protein (PBV); ^a,b^—mean values in the same row with different superscript letters differ significantly at *p* ≤ 0.05; ^A,B^—mean values in the same row with different superscript letters differ significantly at *p* ≤ 0.1.

**Table 4:** Serum biochemical parameters of growing-finishing pigs.

Item	Two-Phase Feeding System				Three-Phase Feeding System			
	Group ^1^							
	C	L	L+AA	*p*-Value	C	L	L+AA	*p*-Value
Reduced levels of crude protein and amino acids (%)	0	15	15 +AA		0	15	15+ AA	
different inclusion levels of crude protein and limiting essential amino acids								
Crude protein (g/L)	65.9	65.1	67.5	0.093	6.69 ^B^	6.60 ^B^	6.39 ^A^	0.007
Urea (g/dL)	29.02 ^a^	27.01 ^b^	24.73 ^c^	0.042	25.44	23.00	24.64	0.198
AST (U/L)	37.75 ^a^	32.88 ^b^	35.38 ^c^	0.036	32.96 ^a^	35.21 ^ab^	37.71 ^b^	0.058
ALT (U/L)	46.20 ^a^	47.75 ^a^	43.31 ^b^	0.051	46.10 ^a^	49.00 ^b^	47.08 ^ac^	0.029
different feeding systems								
Crude Protein (g/dL)	6.62				6.57			0.052
Urea (g/dL)	27.01				24.63			0.333
AST (U/L)	35.34				35.29			0.307
ALT (U/L)	45.75				47.39			0.395

^1^—explanation as in Table 1; ^a,b,c^—mean values in the same row with different superscript letters differ significantly at *p* ≤ 0.05; ^A,B^—mean values in the same row with different superscript letters differ significantly at *p* ≤ 0.01.

**Table 5:** Effect of the inclusion levels of crude protein and limiting essential amino acids in pig diets and feeding system on fecal pH and fecal nitrogen and ammonia concentrations.

Item	Two-Phase Feeding System				Three-Phase Feeding System			
	Group ^1^							
	C	L	L+AA	*p*-Value	C	L	L+AA	*p*-Value
Reduced levels of crude protein and amino acids (%)	0	15	15 +AA		0	15	15+ AA	
different inclusion levels of crude protein and limiting essential amino acids								
Fecal pH	6.40 ^b^	6.16 ^a^	6.29 ^ab^	0.031	6.32	6.17	6.23	0.244
Total fecal N (%)	0.75	0.81	0.73	0.082	0.74	0.74	0.71	0.257
Fecal ammonia (%)	0.04	0.05	0.04	0.857	0.04	0.04	0.05	0.254
different feeding systems								
Fecal pH	6.28				6.24			0.487
Total fecal N (%)	0.77				0.73			0.058
Fecal ammonia (%)	0.04				0.05			0.379

^1^—explanation as in Table 1; ^a,b^—mean values in the same row with different superscript letters differ significantly at *p* ≤ 0.05.

**Table 6:** Effect of the inclusion levels of crude protein and limiting essential amino acids in pig diets and feeding system on urinary pH and urinary nitrogen and ammonia concentrations.

Item	Two-Phase Feeding System				Three-Phase Feeding System			
	Group ^1^							
	C	L	L+AA	*p*-Value	C	L	L+AA	*p*-Value
Reduced levels of crude protein and amino acids (%)	0	15	15 +AA		0	15	15 +AA	
different inclusion levels of crude protein and limiting essential amino acids								
Urinary pH	6.59	6.31	6.46	0.320	6.66 ^B,a^	6.14 ^A,a^	6.41 ^b^	0.005
Total urinary N (%)	0.57	0.58	0.59	0.957	0.52	0.53	0.52	0.761
Urinary ammonia (%)	0.40	0.37	0.40	0.664	0.37	0.40	0.41	0.993
different feeding systems								
Urinary pH	6.45				6.40			0.496
Total urinary N (%)	0.58				0.52			0.125
Urinary ammonia (%)	0.38				0.39			0.868

^1^—explanation as in Table 1; ^a,b^—mean values in the same row with different superscript letters differ significantly at *p* ≤ 0.05; ^A,B^—mean values in the same row with different superscript letters differ significantly at *p* ≤ 0.01.

**Table 7:** Effect of the inclusion levels of crude protein and limiting essential amino acids in pig diets on nitrogen excretion.

Item	Two-Phase Feeding System			Three-Phase Feeding System		
	Group ^1^					
	C	L	L+AA	C	L	L+AA
Reduced levels of crude protein and amino acids (%)	0	15	15 +AA	0	15	15 +AA
N intake (g kg^−1^)	987.7	852.6	847.0	950.1	824.1	816.7
Fecal N excretion (g kg^−1^)	176.4	175.0	150.5	168.9	159.6	149.8
Urinary N excretion (g kg^−1^)	416.5	307.3	308.7	396.7	317.8	320.6
Fecal and urinary N excretion (g kg^−1^)	592.9	482.3	459.2	565.6	477.4	471.4
Reduction in nitrogen excretion depending on:						
15% reduction in dietary inclusion levels of crude protein and limiting essential amino acids:						
(g kg^−1^)	0.0	−110.6	n/a	0.0	−88.2	n/a
(%)	100	81.3	n/a	100	84.4	n/a
Supplementation of low-protein diets with limiting essential amino acids						
(g kg^−1^)	n/a	0.0	−23.1	n/a	0.0	−7.0
(%)	n/a	100	95.2	n/a	100	98.3
Fecal and urinary N excretion in different feeding systems						
	two-phase feeding system			three-phase feeding system		
(g kg^−1^)	511.5			504.5		
Reduction in nitrogen excretion in different feeding systems						
(g kg^−1^) (%)	0.0 100			−7.0 98.6		

^1^—values in the Table are arithmetic means per pig obtained in digestibility-balance trials for grower–finisher diets in the two-phase feeding system and grower 1/grower 2/finisher diets in the three-phase feeding system. n/a—not applicable.

## Data Availability

The data presented in this study are available on request from the corresponding author.
